# Plants Drive Microbial Biomass and Composition but Not Diversity to Promote Ecosystem Multifunctionality in Karst Vegetation Restoration

**DOI:** 10.3390/microorganisms13030590

**Published:** 2025-03-04

**Authors:** Yunlong Sun, Shu Zhang, Yueming Liang, Xuan Yu, Fujing Pan

**Affiliations:** 1 College of Environmental and Engineering, Guangxi Key Laboratory of Environmental Pollution Control Theory and Technology, Guilin University of Technology, Guilin 541006, China; sunyunlong@glut.edu.cn (Y.S.); zhangshu_mail@126.com (S.Z.); 2120220553@glut.edu.cn (X.Y.); 2 Engineering Research Center of Watershed Protection and Green Development for University in Guangxi, Guilin University of Technology, Guilin 541006, China; 3 Karst Dynamics Laboratory, Ministry of Natural Resources, Institute of Karst Geology, Chinese Academy of Geological Sciences, Guilin 541004, China

**Keywords:** karst ecosystem, bacteria, fungi, multifunctionality, vegetation restoration

## Abstract

Natural restoration has emerged as a prominent approach in recent decades for the rehabilitation of degraded ecosystems globally. However, the specific changes and underlying mechanisms by natural restoration that influence the multifunctionality of karst ecosystems remain poorly understood. In this study, soil, litter, and fine root samples were collected from four chronosequence stages of vegetation restoration—grassland (G), shrubland (SH), shrub-tree land (ST), and forest (F)—within a karst ecosystem in Southwestern China. The aim was to evaluate the impacts of vegetation restoration on ecosystem multifunctionality using an averaging approach. The results demonstrated that the indices of C-cycling functionality, N-cycling functionality, P-cycling functionality, and total ecosystem multifunctionality increased as vegetation restoration progressed, along with plant diversity. The structure of plant, bacterial, and fungal communities varied across different stages of vegetation restoration, exhibiting the highest microbial diversity indices in the SH stage. Additionally, the tightness and complexity of co-occurrence networks of bacteria and fungi increased with advancing vegetation restoration, and higher positive links were observed in fungi than bacteria. The four functional indices were significantly and positively correlated with increasing plant diversity, fine root and litter nutrient contents, fine root biomass, microbial biomass, fungal community, enzyme activities, and soil nutrient contents but not with bacterial and fungal diversities. Furthermore, Random Forest model results revealed that plants exerted a significantly greater influence on ecosystem multifunctionality compared to other factors. It is plausible that plants influence soil microbial biomass, fungal community and co-occurrence networks, enzyme activities, and nutrient levels through the input of root and litter nutrients rather than by altering microbial diversity to enhance karst ecosystem multifunctionality. Therefore, initiatives to increase plant diversity are beneficial for sustainable ecological restoration management in the karst regions of Southwestern China.

## 1. Introduction

Ecosystem multifunctionality serves as an integrative indicator reflecting the structural and functional attributes of ecosystems, encompassing the carbon (C)-cycling functional, nitrogen (N)-cycling functional, phosphorus (P)-cycling functional, and total multifunctionality indices [1,2,3,4]. Research has demonstrated that degraded ecosystems typically exhibit lower multifunctionality indices [5], attributed to the loss of aboveground vegetation, diminished belowground microbial diversity, and reduced soil fertility [6,7]. Conversely, undisturbed and restored ecosystems display higher multifunctionality indices [8,9,10], characterized by greater aboveground and belowground biomass [11], enhanced biodiversity [12], more intricate community structures [13], and improved nutrient contents and availabilities in soils [14]. In recent decades, numerous ecosystems have faced significant degradation and destruction globally, such as the Amazon, Southeast Asia, and other regions [15,16,17]. Conversely, China has made substantial progress in restoring vegetation, leading to a notable increase in forest coverage. Notably, the regreening rate of karst ecosystems in Southwestern China has shown rapid improvement [18], attributed to China’s comprehensive ecological restoration initiatives, such as “grain-for-green, afforestation, and rocky desertification control projects” [19]. However, it is important to recognize that karst ecosystems remain inherently fragile due to their unique soil/rock structure [20], which results in substantial water and soil leakage, low nutrient retention, and limited nutrient supply capacity. While the trend of worsening rocky desertification has been effectively mitigated and surface vegetation has improved, challenges related to soil fertility deficiencies persist [19].

Natural restoration, as one of the most cost-effective restoration techniques, has been widely applied in degraded ecosystems globally to enhance soil and water conservation, C and N sequestration, P cycling, and ecosystem services [21,22,23]. In general, natural restoration demonstrates significantly higher levels of soil organic C, N, P, and water content, as well as a notably lower evapotranspiration rate compared to artificial restoration [24]. This phenomenon may be attributed to the more complex stand structure and greater biodiversity associated with natural restoration, leading to increased litter return [25], root exudate input [26,27], and a more diverse chemical composition [28]. In contrast, another study has demonstrated that artificial restoration enhances soil quality (assessed through bulk density, natural water content, total porosity, capillary porosity, and mechanical composition) compared to natural restoration [29]. The ecosystem multifunctionality index can be used to evaluate the benefits of vegetation restoration in sustaining multiple ecosystem functions. Extensive research has consistently shown a strong positive correlation between ecosystem multifunctionality and plant diversity [12,30,31]. It is crucial to acknowledge, however, that plant growth and community structure are influenced by a multitude of factors, including nutrients, water, climate, human disturbance, and others. Previous studies have shown that vegetation in karst regions initially experiences N limitation during the early stages of restoration, followed by the co-limitation of N and P in the intermediate stages, and, ultimately, P limitation in the later stages [32,33]. Nevertheless, there is limited understanding of how ecosystem multifunctionality indices change throughout the vegetation restoration process as nutrient limitation status evolves, as well as the underlying influencing factors and mechanisms.

Vegetation restoration is typically associated with an increase in soil microbial diversity and biomass [34,35,36], as well as alterations in community structure [37]. Soil microorganisms play a crucial role in maintaining or enhancing the soil ecosystem multifunctionality. Recent advancements in research have increasingly focused on elucidating the mechanisms by which soil microorganisms influence the multifunctionality of ecosystems. For instance, vegetation restoration can lead to positive shifts in soil microbial community structure, resulting in a higher ecosystem multifunctionality index linked to a higher complex fungal network but a less complex bacterial network [38,39]. This phenomenon has also been observed in other karst regions, such as Hungary [40]. Additionally, some studies have also demonstrated a positive correlation between a high multifunctionality index and soil microbial diversity [41,42]. Nevertheless, microbial diversity may not consistently serve as a reliable indicator of alterations in ecosystem multifunctionality. The gene abundances, community composition, and biomass of soil microbes provides a more robust explanation for the trends in soil multifunctionality compared to microbial diversity and network complexity [12,43,44]. In regions outside of China’s karst ecosystems and in areas beyond China, several studies have elucidated the contribution of vegetation–soil–microbial relationships to ecosystem multifunctionality [37,45,46]. However, within karst regions, the effects of vegetation restoration on soil ecosystem multifunctionality through plant diversity and nutrient input, as well as the mechanisms by which microbial actions influence soil microbial properties (such as enhancing soil microbial diversity, adjusting microbial community structure, or altering microbial activity), remain relatively understudied. For instance, a previous study has examined the impact of vegetation restoration on karst ecosystem multifunctionality, focusing primarily on the aboveground vegetation community structure, diversity, biomass, and functional traits, thereby neglecting the influence of belowground interactions on ecosystem multifunctionality [47]. Another study has demonstrated that vegetation degradation adversely affects soil microbial communities and diminishes karst ecosystem multifunctionality [48], indicating the potential benefits of vegetation restoration for enhancing ecosystem multifunctionality. Furthermore, a recent article highlighted how artificial restoration measures can significantly improve soil ecosystem multifunctionality in karst farmland areas, with restoration efforts notably impacting the soil microbial community structure and ecosystem multifunctionality [49]. Therefore, a deeper understanding of the impacts of vegetation restoration on ecosystem multifunctionality and the underlying mechanisms is crucial for assessing the effectiveness of natural restoration on the functions and services of karst ecosystems, as well as identifying key factors for sustainable ecological restoration.

To gain a comprehensive understanding of these issues, we conducted an in-depth analysis of four indices of soil ecosystem functions (C-cycling functionality, N-cycling functionality, P-cycling functionality, and total ecosystem multifunctionality) and plant diversity across 20 plots representing four distinct successional stages in a karst ecosystem in Southwestern China. Additionally, we also analyzed soil bacterial and fungal diversity, community structure, and co-occurrence networks and the physicochemical properties. This study aimed to achieve the following: (1) examine the patterns of soil ecosystem multifunctionality and plant diversity across various stages of vegetation restoration in this karst ecosystem; and (2) determine the key drivers influencing soil ecosystem multifunctionality during the process of karst vegetation restoration. The four stages of vegetation restoration examined in this study included grassland (G), shrubland (SH), shrub-tree land (ST), and forest (F). We hypothesized that (1) the indices of soil C-cycling functionality, N-cycling functionality, P-cycling functionality, and total ecosystem multifunctionality would increase as vegetation restoration progresses and plant diversity increases; and (2) plant diversity is the principal driver influencing ecosystem multifunctionality by modulating microbial biomass and community composition, rather than microbial diversity.

## 2. Materials and Methods

### 2.1. Study Site

The study site was selected in a karst ecosystem located in Guilin (24°85′–25°19′ N, 110°37′–110°51′ E), Guangxi Zhuang Autonomous Region, Southwestern China. This site has a mean annual temperature of approximately 19 °C and experiences mean annual precipitation ranging from 1800 to 2000 mm, which are characteristic features of a subtropical monsoon climate [50,51]. Notably, over 65% of the precipitation occurs between May and September. The soils are classified as calcareous lithosols, which are limestone-derived soils, based on the FAO/UNESCO classification system [52].

**Figure 1:**
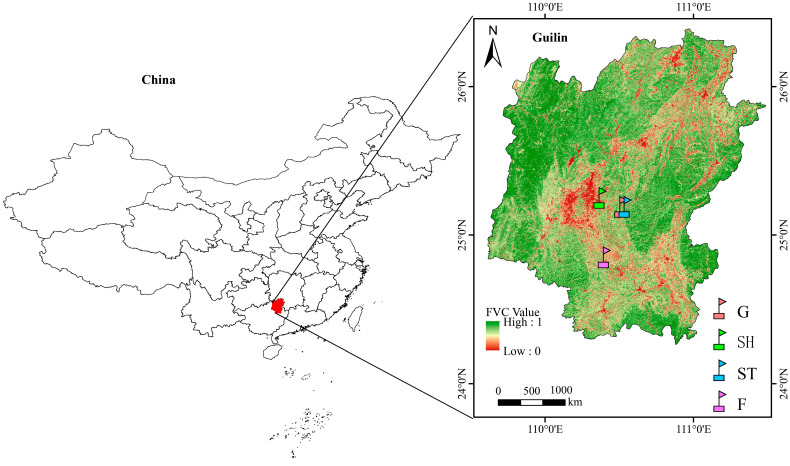
Map of the four stages of vegetation restoration in Guilin, Southwestern China.

In July 2021, four vegetation types (G, SH, ST, and F) were selected as restoration stages based on the space-for-time substitution principle (Figure 1). These vegetations have undergone natural restoration for approximately 15, 30, 45, and 60 years, respectively. At each stage, five replicate plots (each measuring 20 m × 20 m) were established, with a minimum distance of 50 m between adjacent plots. A total of twenty plots were established across all stages. Vegetation surveys were subsequently conducted in these plots to assess the number, height, and canopy coverage of grasses, shrubs, and trees, as well as the diameter at breast height (DBH > 1.5 cm) of trees. Plant diversity was calculated using the Shannon–Wiener, Simpson, and Pielou indices; subsequently, the relative abundance of each plant species was employed to generate graphical representations [32]. Detailed characteristics of these plots, including their dominant species, are provided in Table 1.

### 2.2. Sampling

Soil (0–20 cm depth), fine root (0–20 cm depth), and litter (20 cm × 20 cm) samples were collected from each plot [33]. Five replicate soil cores in each plot were collected using an auger (10 cm diameter) and mixed into a single soil sample. Fine root samples (diameter < 2 mm) were collected using the same methodology as soil sampling and subsequently soaked in water for 24 h to facilitate the removal of adhering soils. Litter samples were also collected with five replicates and mixed into a single sample. A total of 20 samples were collected for soil, fine roots, and litter, respectively. After being ground to pass through a 2 mm mesh sieve, each soil sample was divided into three subsamples by the quartering method. One portion was stored at 4 °C for the determination of soil enzyme activities and microbial biomass. Another portion was stored at −80 °C for the identification of bacterial and fungal diversity and communities. The remaining portion was air-dried, ground, and passed through a 0.85 mm mesh sieve for subsequent physicochemical analysis. Each fine root and litter sample was oven-dried at 65 °C for a minimum of 48 h until reaching constant weight, after which the samples were ground to pass through a 0.154 mm mesh sieve.

### 2.3. Analyses

#### 2.3.1. General Soil, Fine Root, and Litter Parameters

The soil pH was determined using a pH meter at a soil-to-water ratio of 1:2.5. Soil organic carbon (SOC) content was determined through wet oxidation using KCr_2_O_7_ + H_2_SO_4_, followed by titration with FeSO_4_. Total nitrogen (TN) was analyzed using the Kjeldahl method via a FIAstar 5000 flow injection analyzer (FOSS, Hillerød, Denmark) using the Kjeldahl method. Total phosphorus (TP) was measured after digestion in a mixture of H_2_SO_4_ and HClO_4_, followed by colorimetric analysis using the blue phosphor-molybdate method. Ammonium N (NH_4_^+^-N) and nitrate N (NO_3_^−^-N) were extracted from fresh soils using 2 M of KCl and subsequently measured with a FIAstar 5000 flow injection analyzer. Available P (AP) was determined as the total soil P following extraction with NaHCO_3_. Exchangeable calcium (Ca) and magnesium (Mg) were displaced using 1 mol·L^−1^ of ammonium acetate at pH 7.0 and quantified using inductively coupled plasma atomic emission spectroscopy (Optima 7000DV, Perkin Elmer Technologies, Waltham, MA, USA) [32,53]. Microbial biomass C (MBC) was quantified using a total organic carbon analyzer (Shimadzu TOC-Vwp; Shimadzu Corporation, Kyoto, Japan) following extraction with K_2_SO_4_ (0.5 mol·L^−1^). Microbial biomass N (MBN) was assessed using a FIAstar 5000 flow injection analyzer (FOSS, Hillerød, Denmark), while microbial biomass P (MBP) was determined as the AP [54]. The contents of C and N in fine roots and litters were quantified using an elemental analyzer (Vario MAX CN, Elementar, Hanau, Germany). The P contents in fine roots and litter were analyzed after digestion in a mixed acid solution of H_2_SO_4_ + H_2_O_2_, followed by colorimetric determination using the blue phosphor-molybdate method (Spectrophotometer, V-5800, Shanghai Metash Instruments Co., Ltd., Shanghai, China) [55]. The suspensions of β-1-4 glucosidase (βG), β-1,4-N-cetylglucosaminidase (NAG), leucine aminopeptidase (LAP), and acid phosphatase (ACP) were prepared in 50 mM acetate buffer at pH 5. The suspension of alkaline phosphatase (ALP) was prepared in 50 mM sodium bicarbonate buffer at pH 8. Subsequently, the enzyme activities were measured using their respective substrates: 4-methylumbelliferyl-β-D-glucoside for βG, 4-methylumbelliferyl-N-acetyl-β-D-glucosaminide for NAG, L-leucine-7-amido-4-methylcoumarin hydrochloride for LAP, and 4-methylumbelliferyl phosphate for ACP and ALP. Enzyme activities were quantified using a microplate fluorometer (Infinite M200 PRO; TECAN, Männedorf, Switzerland) with excitation at 365 nm and emission at 450 nm [55].

#### 2.3.2. DNA Extraction and Sequencing of Bacteria and Fungi

Genomic DNA from soil bacteria and fungi was extracted from 0.5 g of frozen soil samples using the FastDNA SPIN Kit (MP Biomedicals, Cleveland, OH, USA). The quality and quantity of the extracted DNA were assessed using 1% agarose gel electrophoresis and UV spectrophotometry (NanoDrop Technologies, Wilmington, NC, USA), respectively. For PCR amplification of bacterial 16S rRNA genes, the primers used were 515F (5′-GTGCCAGCMGCCGCGGTAA-3′) and 907R (5′-CCGTCAATTCCTTTGAGTTT-3′) [56]. For fungal ITS genes, the primers were ITS1 (5′-TCCGTAGGTGAACCTGCGC-3′) and ITS4 (5′-TCCTCCGCTTATTGATATGC-3′) [57].

The reaction was performed in triplicate with a total volume of 25 μL per sample, comprising 2.5 μL of 10 × Ex Taq Buffer (containing Mg^2+^), 0.3 μL of Ex Taq polymerase (Takara, Otsu, Shiga, Japan), 1 μL of each forward and reverse primer (10 pM), 1 μL of DNA template (~30 ng), and ddH_2_O added to a final volume of 25 μL. The amplification protocol for bacteria comprised an initial denaturation at 95 °C for 3 min, followed by 30 cycles of denaturation at 95 °C for 30 s, annealing at 50 °C for 30 s, and extension at 72 °C for 30 s, concluding with a final elongation step at 72 °C for 10 min [56]. For the fungal samples, the amplification protocol comprised an initial denaturation at 95 °C for 3 min, followed by 20 cycles consisting of denaturation at 95 °C for 30 s, annealing at 55 °C for 30 s, and extension at 72 °C for 1 min [57].

The bacterial polymerase chain reaction (PCR) products were purified using the TianGen TIANquick Midi Purification Kit (from China TianGen, Beijing, China). For fungi, the GeneTools analysis software (version 4.03.05.0, SynGene, Frederick, MD, USA) was used to compare the concentrations of the PCR products. Based on the principle of equal mass, the volume required for each sample was calculated, and the PCR products were mixed accordingly. The E.Z.N.A.^®^ Gel Extraction Kit (Omega, GA, USA) was used to recover the PCR mixed products, and the target DNA fragments were eluted with TE buffer. The library construction was carried out following the standard protocol of the NEBNext^®^ Ultra™ DNA Library Prep Kit, and the sequencing was performed on the Illumina HiSeq2500 platform (Guangdong Magigene Biotechnology Co., Ltd., Guangzhou, China). The BioProject accession numbers for the sequence data deposited in the NCBI database are PRJNA1212538 for bacterial sequences and PRJNA1212499 for fungal sequences.

### 2.4. Data Analyses

#### 2.4.1. Co-Occurrence Networks of Bacteria and Fungi

The observed OTUs of bacteria and fungi that were present in less than 0.1% of our present samples were excluded to minimize potential biases during network construction. Spearman’s correlation coefficients among the selected OTUs were computed using the “cor.test” function in the “psych” package (version 2.1.3) of R [58]. Correlations with an r-value greater than 0.6 and a *p*-value less than 0.05, after Bonferroni correction, were utilized to construct co-occurrence networks for each stage of vegetation restoration using Gephi version 0.9 [53].

#### 2.4.2. Calculation of Multifunctionality Index

In this study, we quantified the functional indices of C-cycling, N-cycling, and P-cycling, as well as the total multifunctionality index, for various vegetation restorations in a karst ecosystem using an averaging approach [59]. Specifically, the C-cycling functional index was calculated based on βG activity and the contents of SOC, MBC, and fine root and litter C. The N-cycling functional index was determined by the activities of NAG and LAP, along with the contents of TN, NH_4_^+^-N, NO_3_^−^-N, MBN, and fine root and litter N. For the P-cycling functional index, we considered the activities of ACP and ALP, as well as the contents of TP, AP, MBP, and fine root and litter P. A total of 20 parameters was used to calculate the total multifunctionality index. These parameters were first standardized using Z-score transformation and then averaged to derive the multifunctionality index for each plot, as well as the C-cycling, N-cycling, and P-cycling functional indices. These indices were subsequently utilized to assess the variations among different vegetation restorations.

#### 2.4.3. Statistical Analyses

The normality of distribution and homogeneity of variance were evaluated for all parameters examined in this study. Two-way analysis of variance (ANOVA) was conducted at a significance level of *p* < 0.05 to evaluate differences in C-cycling, N-cycling, and P-cycling functional indices, as well as total multifunctionality indices, among the four vegetation types. Additionally, ANOVA was employed to analyze plant, bacterial, and fungal diversity indices. Furthermore, ANOVA was used to analyze soil nutrients, fine root nutrients, litter nutrients, microbial biomass contents, and enzyme activities. Subsequently, differences in plant, bacterial, and fungal communities across the four vegetation restoration stages were assessed using non-metric multidimensional scaling (NMDS) with the “vegan” package in R version 4.4.0 [58]. The NMDS analysis codes are provided in the Supplementary File.

The correlations of C-cycling, N-cycling, and P-cycling functional indices and total multifunctionality indices with respect to soil nutrients, fine root nutrients, litter nutrients, microbial biomass and community, and enzyme activities were quantitatively visualized using the Pearson analysis with the “corrplot”, “vegan”, and “ggplot2” packages in R version 4.4.0. In addition, the relationships among the soil nutrients, fine root nutrients, litter nutrients, microbial biomass contents and enzyme activities were also visually calculated (Figure S1). Ultimately, a Random Forest model was employed to assess the relative importance of each plant, soil, and microbial parameter in relation to C-cycling, N-cycling, and P-cycling functional indices, as well as total multifunctionality indices. This evaluation was conducted using the “randomForest” package in R, with the relative importance of the measured variables quantified by the mean square error (MSE %).

## 3. Results

### 3.1. Patterns of Soil, Root, and Litter Nutrients, Enzyme Activities, and Ecosystem Multifunctionality

**Figure 2:**
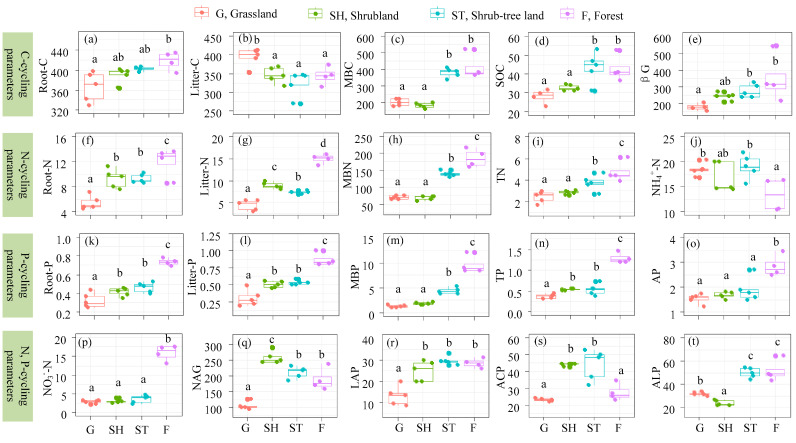
Soil, fine root, litter nutrient and enzyme activities across advancing vegetation restorations. C-cycling parameters: Root-C (**a**), C contents of fine root; Litter-C (**b**), C contents of litter; MBC (**c**), microbial biomass C; SOC (**d**), soil organic carbon; and βG (**e**), β-Glucosidase activity. N-cycling parameters: Root-N (**f**), N contents of fine root; Litter-N (**g**), N contents of litter; MBN (**h**), microbial biomass N; TN (**i**), soil total N; NH_4_^+^-N (**j**), ammonium N; NO_3_^−^-N (**p**), nitrate N; NAG (**q**), β-1,4-N-acetylglucosaminidase activity; and LAP (**r**), leucine aminopeptidase activity. P-cycling parameters: Root-P (**k**), P contents of fine root; Litter-P (**l**), P contents of litter; MBP (**m**), microbial biomass P; TP (**n**), soil total P; AP (**o**), soil available P; ACP (**s**), acid phosphatase; ALP (**t**), alkaline phosphatase; and grassland (G), shrubland (SH), shrub-tree land (ST), and forest (F). Different letters mean significant differences (*p* < 0.05).

The contents of C, N, P in fine roots and microbial biomass, as well as N and P in litters, and SOC, TN, NO_3_^−^-N, TP, AP, and the activities of βG, LAP, and ALP in soils, all increased with the progression of vegetation restoration (Figure 2). Conversely, the contents of C in litters and NH_4_^+^-N in soils decreased as vegetation restoration advanced. The activities of NAG and ACP increased from G to SH and ST but then declined in F.

**Figure 3:**
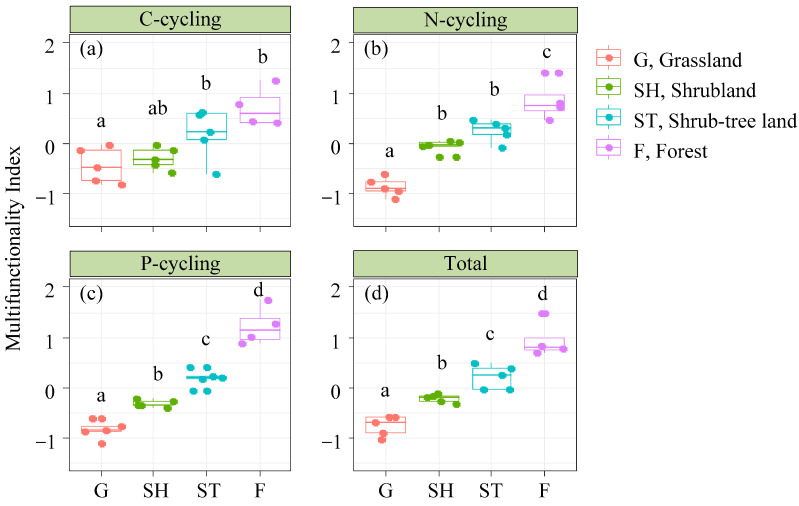
The patterns of C-cycling functional (**a**), N-cycling functional (**b**), P-cycling functional (**c**), and total multifunctionality (**d**) indices across advancing vegetation restorations. Different letters mean significant differences (*p* < 0.05).

Indices of C-cycling functionality, N-cycling functionality, P-cycling functionality, and total multifunctionality indices increased progressively with the advancement of karst vegetation restoration (Figure 3).

### 3.2. Diversity Indices of Plants, Bacteria, and Fungi and Microbial Community Structure

**Figure 4:**
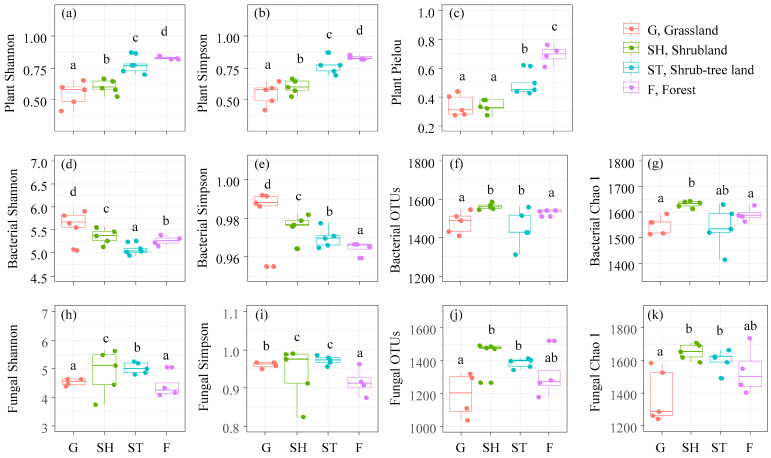
The diversity indices of plants, bacteria, and fungi across advancing vegetation restorations. Plant Shannon (**a**), the Shannon–Wiener index of plants; Plant Simpson (**b**), the Simpson index of plants; Plant Pielou (**c**), the Pielou index of plants; bacterial Shannon (**d**), the Shannon–Wiener index of bacteria; bacterial Simpson (**e**), the Simpson index of bacteria; bacterial OTUs (**f**); bacterial Chao1 (**g**); fungal Shannon (**h**), the Shannon–Wiener index of fungi; and fungal Simpson (**i**), the Simpson index of fungi; fungi OTUs (**j**); fungi Chao1 (**k**). Different letters mean significant differences (*p* < 0.05).

**Figure 5:**
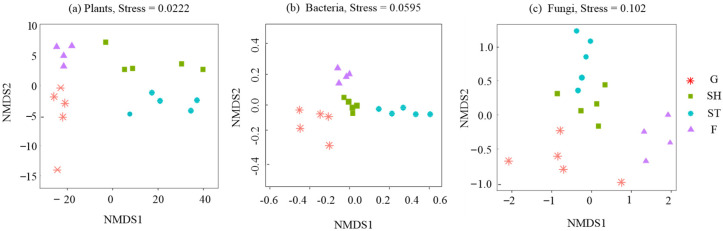
Community structures of plants, bacteria, and fungi across advancing vegetation restorations.

The Shannon–Wiener, Sampson, and Pielou indices for plant diversity increased progressively with the advancement of karst vegetation restoration (Figure 4a–c). Conversely, the Shannon–Wiener and Simpson indices for bacterial diversity exhibited a declining trend (Figure 4d,e). The observed OTUs and Chao 1 indices of bacteria increased from G to SH and then decreased to F (Figure 4f,g). Similar patterns were observed for fungal diversity, where the Shannon–Wiener, Simpson, OTUs, and Chao 1 indices peaked in SH (Figure 4h–k). Furthermore, the community structures of plants, bacteria, and fungi exhibited significant variations across different stages of vegetation restoration (Figure 5a–c).

**Figure 6:**
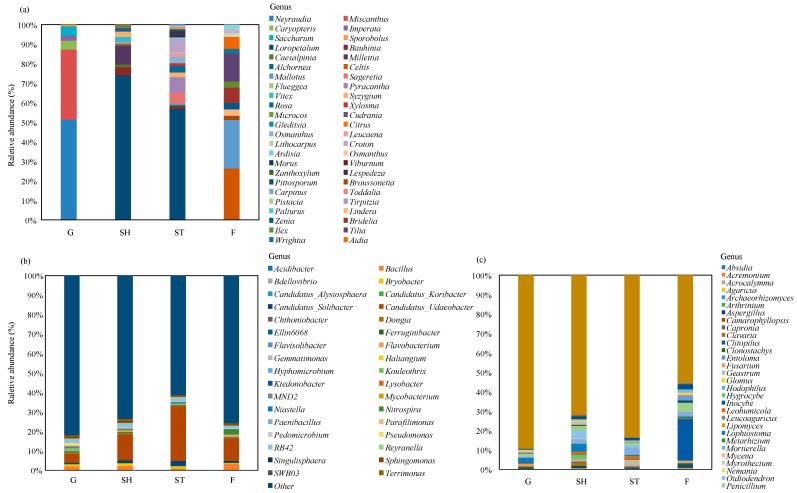
Relative abundances at genus level of plants (**a**), bacteria (**b**), and fungi (**c**) across advancing vegetation restorations.

For plants, at the genus level, *Neyraudia* (51.36%) and *Miscanthus* (35.92%) were predominant in G; *Loropetalum* (74.02% and 57.04%, respectively) dominated in SH and ST; and *Celtis* (18.36%), *Mallotus* (17.35%), and *Tilia* (10.13%) were predominant in F (Figure 6a). At the order level, Poales (95.47%) was predominant in G, Hamamelidales (74.02% and 57.04%, respectively) in SH and ST, and Malpighiales (28.05%) and Fagales (26.29%) in F (Figure S2a).

For bacteria, at the genus level, *Candidatus_Udaeobacter* (4.27%, 13.08%, 27.85%, and 11.50%, respectively) was predominant in G, SH, ST, and F (Figure 6b). At the order level, Chthoniobacterales (5.46%, 15.79%, 32.70%, and 15.14%, respectively), Myxococcales (8.43%, 15.86%, 5.74%, and 19.71%, respectively), and Rhizobiales (6.13%, 7.91%, 7.31%, and 6.56%, respectively) were predominant in G, SH, ST, and F (Figure S2b). At the phylum level, Proteobacteria (31.05%), Acidobacteria (30.64%), and Actinobacteria (8.28%) were predominant in G, while Proteobacteria (33.32%, 23.99%, and 18.18%, respectively), Acidobacteria (20.27%, 24.55%, and 34.48%, respectively), and Verrucomicrobia (35.23%, 20.20%, and 17.54%, respectively) were predominant in SH, ST, and F (Figure S2c).

For fungi, at the genus level, *Lophiostoma* (2.69%) and *Fusarium* (1.16%) were predominant in G; *Oidiodendron* (3.77%) and *Lophiostoma* (3.51%) in SH; *Mortierella* (3.7%) and *Geastrum* (2.27%) in ST; and *Inocybe* (21.36%) in F (Figure 6c). At the order level, Agaricales (16.88%, 8.38%, and 29.75%, respectively) were predominant in G, ST, and F, while Hypocreales (12.47%) and Pleosporales (11.37%) were predominant in SH (Figure S2d). At the phylum level, Ascomycota (65.32%, 71.63%, and 73.80%, respectively) were predominant in G, SH, and ST, while Basidiomycota (47.66%) and Ascomycota (46.23%) were predominant in F (Figure S2e).

**Figure 7:**
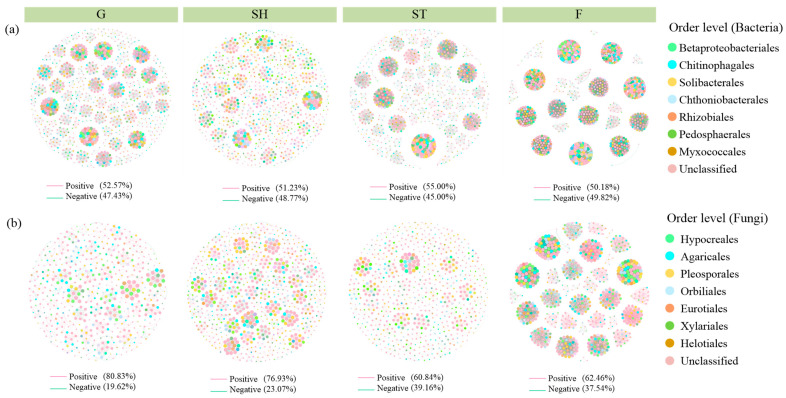
Co-occurrence network models of bacteria (**a**) and fungi (**b**) at order level across advancing vegetation restorations. The red line indicates positive relation, and the green line indicates negative relation.

The density and number of nodes and edges in bacterial and fungal co-occurrence networks increased with advancing vegetation restoration; moreover, higher positive links were observed in fungi than bacteria (Figure 7, Table 2).

### 3.3. Ecosystem Multifunctionality in Relation to Plant, Microbial, and Soil Factors

**Figure 8:**
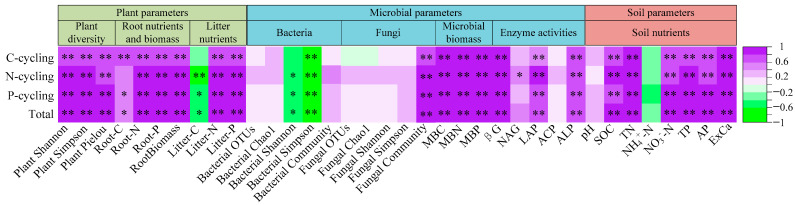
Ecosystem multifunctionality indices related to plant, microbial, and soil factors. C-cycling, C-cycling functional index; N-cycling, N-cycling functional index; P-cycling, P-cycling functional index; Total, total multifunctionality index. Plant Shannon, Shannon–Wiener index of plants; Plant Simpson, Simpson index of plants; Plant Pielou, Pielou index of plants; bacterial Shannon, Shannon–Wiener index of bacteria; bacterial Simpson, Simpson index of bacteria; fungal Shannon, Shannon–Wiener index of fungi; and fungal Simpson, Simpson index of fungi. Root-C, C contents of fine root; Litter-C, C contents of litter; MBC, microbial biomass C; SOC, soil organic carbon; and βG, β-Glucosidase activity. Root-N, N contents of fine root; Litter-N, N contents of litter; MBN, microbial biomass N; TN, soil total N; NH_4_^+^-N, ammonium N; NO_3_^−^-N, nitrate N; NAG, β-1,4-N-acetylglucosaminidase activity; and LAP, leucine aminopeptidase activity. Root-P, P contents of fine root; Litter-P, P contents of litter; MBP, microbial biomass P; TP, soil total P; AP, soil available P; ACP, acid phosphatase; and ALP, alkaline phosphatase. ** *p* < 0.01; * *p* < 0.05.

The C-cycling functional, N-cycling functional, P-cycling functional, and total multifunctionality indices exhibited significant positive correlations with plant diversity (Shannon–Wiener, Simpson, and Pielou indices), fungal community composition, fine root biomass and nutrient contents (C, N, P), microbial biomass C, N, and P, enzymatic activities of βG, LAP, and ALP, as well as SOC, TN, NO_3_^−^-N, TP, AP, and exchangeable Ca (Figure 8). In contrast, these four indices showed significant negative correlations with litter C content and bacterial diversity (Shannon–Wiener and Simpson indices) while exhibiting weak negative relationships with NH_4_^+^-N.

**Figure 9:**
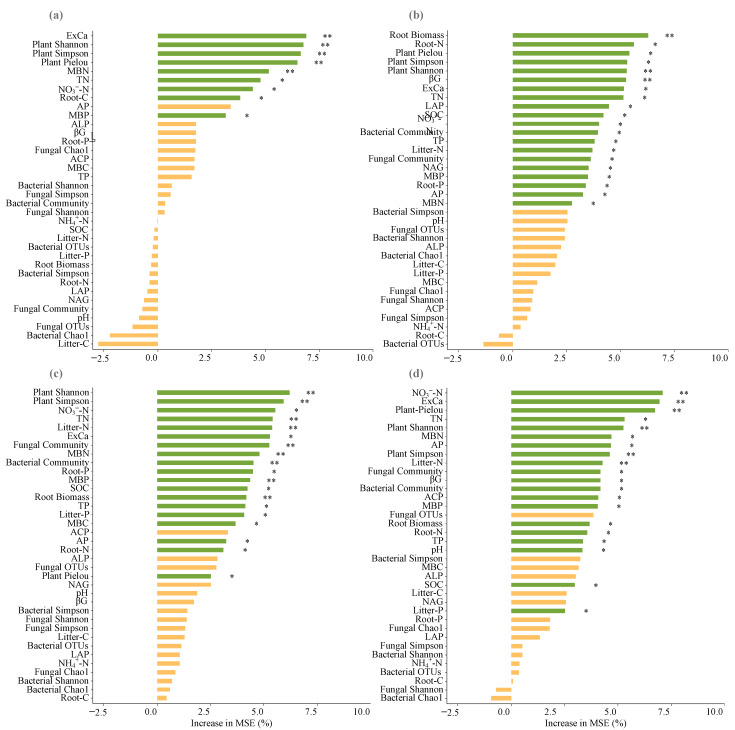
The changes in C-cycling functional (**a**), N-cycling functional (**b**), P-cycling functional (**c**), and total multifunctionality (**d**) indices are importantly ranked by plant, microbial, and soil factors. MSE is the mean square error, and the percentage of variations in MSE is used to estimate the relative importance of the measured variables. Green indicates the significant explanatory variables, while yellow denotes the non-significant explanatory variables. ** *p* < 0.01; * *p* < 0.05.

Random Forest model results showed that changes in C-cycling functional indices were predominantly and significantly predicted by plant parameters (diversity and fine root C contents), microbial biomass (MBN and MBP), and soil nutrients (exchangeable Ca, total N, and NO_3_^−^-N) (Figure 9a). Changes in N-cycling functional indices were primarily and significantly predicted by a broader set of factors, including plant diversity, N and P contents and biomass in fine roots, N content in litters, microbial biomass (MBN and MBP), fungal and bacterial community, soil nutrients (exchangeable Ca, SOC, total N, NO_3_^−^-N, total P, and available P), and enzyme activities (βG, LAP, and NAG) (Figure 9b). For P-cycling functional indices, significant predictors included plant parameters (diversity, N and P contents and biomass in fine roots, and N and P contents in litters), microbial biomass (MBC, MBN, and MBP), fungal and bacterial community, soil nutrients (exchangeable Ca, SOC, total N, total P, NO_3_^−^-N, and available P), and enzyme activities (NAG and LAP) (Figure 9c). The changes in total multifunctionality indices were significantly predicted by plant parameters (diversity, N content and biomass in fine roots, and N and P contents in litters), microbial biomass (MBN and MBP), fungal and bacterial community, soil nutrients (NO_3_^−^-N, exchangeable Ca, total N, AP, total P, and SOC), and enzyme activities (βG and ACP) (Figure 9d). Consequently, the observed enhancement in C-cycling, N-cycling, P-cycling functional indices, and total multifunctionality indices can be attributed to increased plant diversity, nutrient contents in fine roots and litter, fine root biomass, microbial biomass, enzyme activities, and soil nutrients.

## 4. Discussion

### 4.1. Ecosystem Multifunctionality in Advancing Karst Vegetation and Related to Plant, Soil, and Microbial Factors

In this study, we observed a significant increase in the indices of C-cycling functionality, N-cycling functionality, P-cycling functionality, and total multifunctionality as karst vegetation restoration progressed. This suggests that the restoration efforts have effectively enhanced the multifunctionality of the karst ecosystem, thereby supporting our first hypothesis. The enhancement of ecosystem multifunctionality through vegetation restoration is well documented and is closely associated with improvements in plant diversity, soil fertility, and microbial biomass and composition [12,30,31,42,43]. Our findings align with these observations, demonstrating significant positive correlations between the four functional indices and plant diversity, nutrient contents in roots, litter, and soils, microbial biomass, and enzyme activities (Figure 5).

Positive relationships between multifunctionality and plant diversity were clearly demonstrated in this study, consistent with findings from several previous studies. Several potential explanations can account for this phenomenon: (1) The increasing plant diversity enhances coexistence for multiple plant and microbial species. While higher species coexistence can potentially lead to niche competition, these species may evolve convergent resource acquisition strategies, particularly in ecosystems limited by nutrients. Our previous research also demonstrated that plants tend to develop P-exploitative strategies in later stages of restoration when P limitation becomes more pronounced [33]. In response to P limitation, organisms of the same functional type exhibit enhanced cooperation. For instance, the complexity and connectivity of *phoD*-harboring bacterial co-occurrence networks were significantly elevated during vegetation restoration [53]. Similar trends were corroborated by the tightness and complexity of bacterial and fungal co-occurrence networks in our current study, particularly those observed in fungal networks (Figure 6). (2) High plant diversity significantly enhances the availability of water and nutrient resources. In karst regions, a unique geological drought occurs due to the rapid vertical percolation of rainwater through underground karst caves [20]. Increasing plant diversity along vegetation restorations will increase root biomass and litter accumulation, thereby improving soil water retention capacity. Moreover, higher quality root exudates, and more efficient litter decomposition can enhance soil fertility. This is supported by the observed increases in root biomass, litterfall, and soil nutrients in this study, as well as our previous research demonstrating greater secretion of root organic acids in later stages of vegetation restoration [27].

Microorganisms play a crucial role in soil C-, N-, and P-cycling. They secrete hydrolytic enzymes, thereby facilitating the decomposition of organic matter and the transformation of N and P nutrients in soils [60]. In this study, we observed significant positive correlations between the C-cycling functional, N-cycling functional, P-cycling functional, and total multifunctionality indices and the soil microbial biomass, fungal community, and enzyme activities. However, these indices were not significantly correlated with fungal diversity but exhibited negative correlations with bacterial Shannon–Wiener and Simpson indices. Although it was difficult to understand that soil bacterial and fungal diversity did not increase with vegetation restoration, we found significant differences in bacterial and fungal communities between different restoration stages. Numerous studies have demonstrated that vegetation restoration significantly enhances microbial diversity and alters the composition of microbial communities, thereby influencing key indicators of soil ecosystem multifunctionality [35,36,37,38]. Microbial communities may respond to environmental changes through alterations in community structure, the complexity and connectivity of co-occurrence networks, and shifts in biomass rather than their diversities [39,41,53,61]. Based on these findings, it can be concluded that plants play a pivotal role in driving changes in soil ecosystem multifunctionality through the regulation of microbial community structures and co-occurrence networks, with particular emphasis on fungi, during the process of karst vegetation restoration.

**Figure 10:**
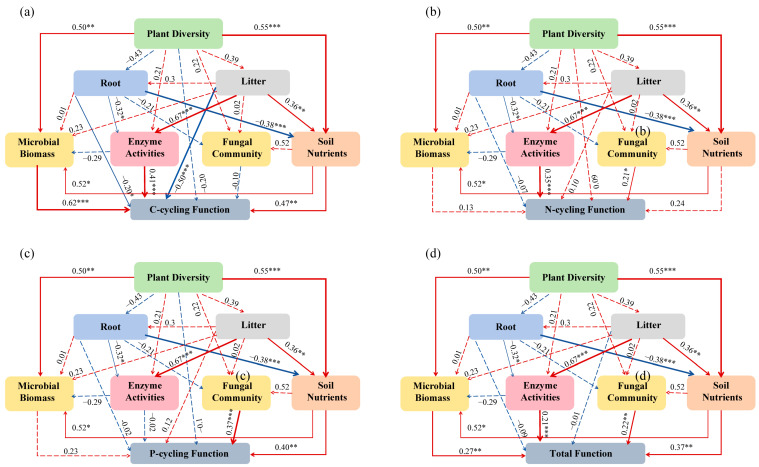
Structural equation model results show that the influence path on soil C-cycling functional (**a**), N-cycling functional (**b**), P-cycling functional (**c**), and total multifunctional (**d**) indices. The parameters of these models: (**a**) X_2_ = 1.741, degrees of freedom = 3, n = 19, CFI = 0.977, AGFI = 0.724, *p* = 0.628, RMSEA = 0.000; (**b**) X_2_ = 1.741, degrees of freedom = 3, n = 19, CFI = 0.977, AGFI = 0.724, *p* = 0.628, RMSEA = 0.000; (**c**) X_2_ = 1.741, degrees of freedom = 3, n = 19, CFI = 0.977, AGFI = 0.724, *p* = 0.628, RMSEA = 0.000; and (**d**) X_2_ = 1.798, degrees of freedom = 4, n = 19, CFI = 0.976, AGFI = 0.786, *p* = 0.773, RMSEA = 0.000. Blue represents negative impact, red represents positive impact. ---, *p* ≥ 0.05, * *p* < 0.05, ** *p* < 0.01, *** *p* < 0.001.

It is important to highlight that, in this study, plants exert a more substantial influence on ecosystem multifunctionality compared to microorganisms (Figure 9), supporting our second hypothesis. Plants impact soil nutrient levels, microbial biomass, fungal community structures and co-occurrence networks, and enzyme activities through modifications in root systems and litter composition [62]. Nevertheless, the present data revealed a negative correlation between litter C content and soil nutrients, microbial biomass, and enzyme activities (Figure S1). In contrast, root nutrient contents and biomass exhibited significant positive correlations with these parameters. The above findings suggest that plant roots exert a more direct and significant influence on karst soil biochemical indicators compared to litter, which is consistent with one previous study [63]. Our current results demonstrate that plants influence soil nutrient content, microbial biomass, and enzyme activities via their roots and litter, thereby enhancing C-cycling function. In addition to these effects, plants also reshaped the fungal community structure and increased fungal co-occurrence network tightness and complexity, which further promotes N-cycling, P-cycling, and total ecosystem multifunctionality (Figure 10).

Proteobacteria (with dominant orders Myxococcales and Rhizobiales and dominant genus *Pseudomonas*), Acidobacteria (with dominant genus *Candidatus_Koribacter* and *Candidatus_Solibacter*), Actinobacteria (with dominant genus *Mycobacterium*), and Verrucomicrobia (with dominant order Chthoniobacterales and dominant genus *Candidatus_Udaeobacter*) were identified as the most dominant bacterial phyla with the advancing vegetation restoration in this karst ecosystem. This pattern was consistently observed not only at another site in Southwest China [64] but also in other calcareous regions, such as the Mediterranean area [65], and in areas undergoing revegetation management [66]. The elevated calcium content in the soil facilitates the survival and reproduction of Proteobacteria [67,68,69]. In addition, the relative abundance of Proteobacteria ranged from 33.32% to 18.18%, showing a gradual decline with advancing vegetation restoration, a trend consistent with findings from another study [70]. Given the close association between Proteobacteria and Rhizobiales [71], this decline suggests a reduction in nitrogen fixation by soil microorganisms as vegetation restoration progresses, likely due to the saturation of soil nitrogen content [72]. The relative abundance of Acidobacteria varied from 20.27% to 34.48%, initially decreasing from grassland to shrubland before gradually increasing in forest. Additionally, while Actinobacteria dominated in grassland, Verrucomicrobia became more prominent in shrubland, shrub-tree land, and forest. Actinobacteria exhibit a competitive advantage in carbon utilization [70], whereas the increase in Verrucomicrobia correlates significantly with plant root growths [73]. Our data also demonstrate a progressive increase in root biomass alongside vegetation restoration, indicating that the rise in Verrucomicrobia is a significant contributor to the enhancement of soil ecosystem multifunctionality. Furthermore, Ascomycota and Basidiomycota were the predominant fungal phyla during vegetation restoration progresses in karst ecosystems, which is consistent with similar observations in Pakistan and Mediterranean restoring forests [74,75,76]. These fungi are recognized for their substantial contributions to soil C-, N-, and P-cycling [70,77]. These above results implied that the dominant bacteria and fungi are deeply involved in soil C-, N-, and P-cycling. Therefore, in this karst ecosystem, plants influence microbial biomass and composition, rather than diversity, to enhance ecosystem multifunctionality with advancing vegetation restoration.

### 4.2. Implications for Vegetation Restoration in Karst Ecosystem

Since the implementation of large-scale ecological projects, a variety of initiatives, including the grain-for-green program, afforestation efforts, and soil erosion control measures, have been applied to fragile karst ecosystems [19]. Based on the principle of ecosystem multifunctionality, vegetation restoration necessitates a more detailed and rigorous methodology through evaluating appropriate parameters [78]. There are two principal reasons for this requirement: firstly, restoration efforts in fragile ecosystems must prioritize regulatory and service functions; and, secondly, achieving rapid restoration while enhancing ecosystem stability is a critical objective. Furthermore, the positive feedback mechanisms between aboveground and belowground processes significantly enhance ecosystem multifunctionality [79]. Our current study reveals that, as vegetation restoration advances, ecosystem multifunctionality exhibits a significant positive correlation with plant diversity, microbial biomass, fungal community composition, enzymatic activities, and soil nutrient levels. The karst ecosystem in Southwestern China has emerged as one of the most rapidly greening regions globally, thereby enhancing the region’s capacity for C sequestration and water regulation [18]. Greater plant diversity contributes increased litter and root-derived nutrients to the soil, which positively influences ecosystem multifunctionality and stability through its effects on both aboveground and belowground feedback mechanisms. Although the ecosystem multifunctionality index is a critical indicator for guiding restoration and management decisions, more detailed data on individual ecosystem components deserve heightened attention. Previous studies have documented a relatively rapid restoration rate of karst vegetation, whereas the restoration of soil fertility has progressed at a slower pace in comparison [18,19]. This asynchronous phenomenon is particularly pronounced in the widespread P deficiency that limits plant growth during the later stages of vegetation restoration in karst ecosystems.

Acquiring comprehensive and large-scale data on vegetation restoration is essential for the effective design and management of restoration initiatives across diverse karst regions. In our previous research, we observed that karst ecosystems at lower latitudes exhibit reduced soil available P content, which is likely attributable to elevated levels of bound P fractions [61]. This condition results in more pronounced P limitations on vegetation restoration in these regions. Large sample analysis can provide detailed insights into the soil and vegetation restoration characteristics across various karst regions, facilitating the development of tailored restoration programs, the precise delineation of forest function zones, and the effective protection of biodiversity. Mitigating the influence of limiting factors can enhance ecosystem resilience, provided that a comprehensive analysis of various factors is conducted prior to the initiation of vegetation restoration projects. Currently, phenomena including droughts, rising temperatures, and increased carbon dioxide concentrations pose increasingly significant challenges to the global ecological environment [80,81]. This study was conducted within a karst ecosystem, where an extensive analysis of various plant, soil, and microbial parameters was performed. It is important to acknowledge that the findings may have limited generalizability beyond the specific study area due to constraints in spatial scale and sample size. To enhance the robustness and applicability of the results, future research should aim for broader spatial coverage and a larger sample size. Additionally, it is recommended that future studies investigate specific locations and scenarios to optimize vegetation restoration methods and management practices at the regional or national level, thereby enhancing the resilience of karst ecosystems and mitigating the impacts of global change.

## 5. Conclusions

We analyze the trends in ecosystem multifunctionality during karst vegetation restoration and elucidate the mechanisms by which plant diversity influences ecosystem multifunctionality through root, litter, and soil dynamics. The C-cycling functional, N-cycling functional, P-cycling functional, and total multifunctionality indices progressively increased with advancing vegetation restoration, coinciding with an increase in plant diversity. These four indices were enhanced by rising plant diversity, nutrient contents of fine roots and litters, fine root biomass, microbial biomass, enzyme activities, and soil nutrients. Furthermore, the structure of bacterial and fungal communities varied across different stages of vegetation restoration. The co-occurrence networks of bacteria and fungi became tighter and more complex as vegetation restoration progressed, with a higher number of positive links observed in fungal communities compared to bacterial communities. It is evident that plants drive microbial biomass and composition, but not diversity, to promote ecosystem multifunctionality in this karst ecosystem. Therefore, preserving plant diversity and fungal community, while optimizing their functional interactions, is an effective strategy to advance karst ecological restoration and enhance ecosystem multifunctionality.

## Figures and Tables

**Table 1:** Plot characteristics of four vegetation restoration stages.

Stages	Altitude (Masl)	Slope (Degrees)	Community Height (m)	Dominant Species
G	194–225	23.2 ± 6.8	2.07 ± 0.72	*Neyraudia reynaudiana* (kunth.) Keng (Dominant), *Miscanthus floridulus* (Lab.) Warb. ex Schum et Laut. (Dominant)
SH	250–282	44.5 ± 5.6	2.33 ± 0.69	*Loropetalum Chinense* (R. Br.) Oliver (Evergreen), *Millettia pulchra* (Benth.) Kurz (N-fixing species)*, Bauhinia championii* (Benth.) Benth. (N-fixing species), *Gleditsia sinensis Lam.* (N-fixing species)
ST	212–244	38.5 ± 6.2	2.64 ± 1.04	*Loropetalum chinense* (R. Br.) Oliver (Evergreen), *Pyracantha fortuneana* (Maxim.) Li (Deciduous), *Sageretia rugosa* Hance (Deciduous)
F	255–297	35.8 ± 4.5	8.40 ± 3.38	*Quercus glauca* Thunb. (Evergreen), *Aidia cochinchinensis* Lour. (Deciduous), *Tilia tuan* Szyszyl. (Deciduous)

**Table 2:** Model parameters of bacterial and fungal co-occurrence networks.

Model Parameters	Bacteria				Fungi			
	G	SH	ST	F	G	SH	ST	F
Average degree	15.029	12.016	21.568	71.602	4.937	7.353	6.767	30.406
Density	0.015	0.014	0.027	0.072	0.01	0.01	0.01	0.044
Modularity	0.958	0.965	0.845	0.878	0.974	0.976	0.965	0.923
Nodes	974	859	806	991	510	764	683	695
Edges	7324	5161	8692	35,479	1259	2809	2311	10,566
Positive links	52.57%	51.23%	55%	50.18%	80.38%	76.93%	60.84%	62.46%
Negative links	47.43%	48.77%	45%	49.82%	19.62%	23.07%	39.16%	37.54%

## Data Availability

The raw data supporting the conclusions of this article will be made available by the authors upon request.

## Supplementary Materials

The following supporting information can be downloaded at https://www.mdpi.com/article/10.3390/microorganisms13030590/s1, Figure S1: Relative abundances at order level of soil bacteria and fungi in advancing vegetation restorations; Figure S2: Soil nutrients, microbial biomass, and enzyme activities related to plant parameters. ** *p* < 0.01; * *p* < 0.05; Figure S3: SEM results showed the direct and indirect effects of factors on soil C-cycling functional (a), N-cycling functional (b), P-cycling functional (c), and total multifunctional (d) indices; and Table S1: Soil pH, soil and litter nutrients, and enzyme activities in advancing vegetation restorations (0–20 cm).
