# Supplementary material for: Plants Drive Microbial Biomass and Composition but Not Diversity to Promote Ecosystem Multifunctionality in Karst Vegetation Restoration
Source: Microorganisms. 2025 Mar 4;13(3):590. doi: 10.3390/microorganisms13030590 (PMC11945124; doi:10.3390/microorganisms13030590)
Supplement: Supplementary file 1 [file microorganisms-13-00590-s001.zip › microorganisms-3490333-supplementary.pdf]

Supplementary materials

The contents of SOC, TN, NO_3-N, TP, AP, and exchangeable Ca were significantly and positively related to plant diversity (Shannon-Wiener, Simpson, Pielou indices), the contents of C, N, and P and biomass in fine roots, the contents of N and P in litters, the microbial biomass C, N, and P, and enzymes activities. The microbial biomass C, N, and P were also significantly and positively related to plant diversity, root and litter nutrients. Additionally, the enzymes activities were also significantly and positively correlated with plant diversity, root and litter nutrients, and microbial biomass (Fig.S1).

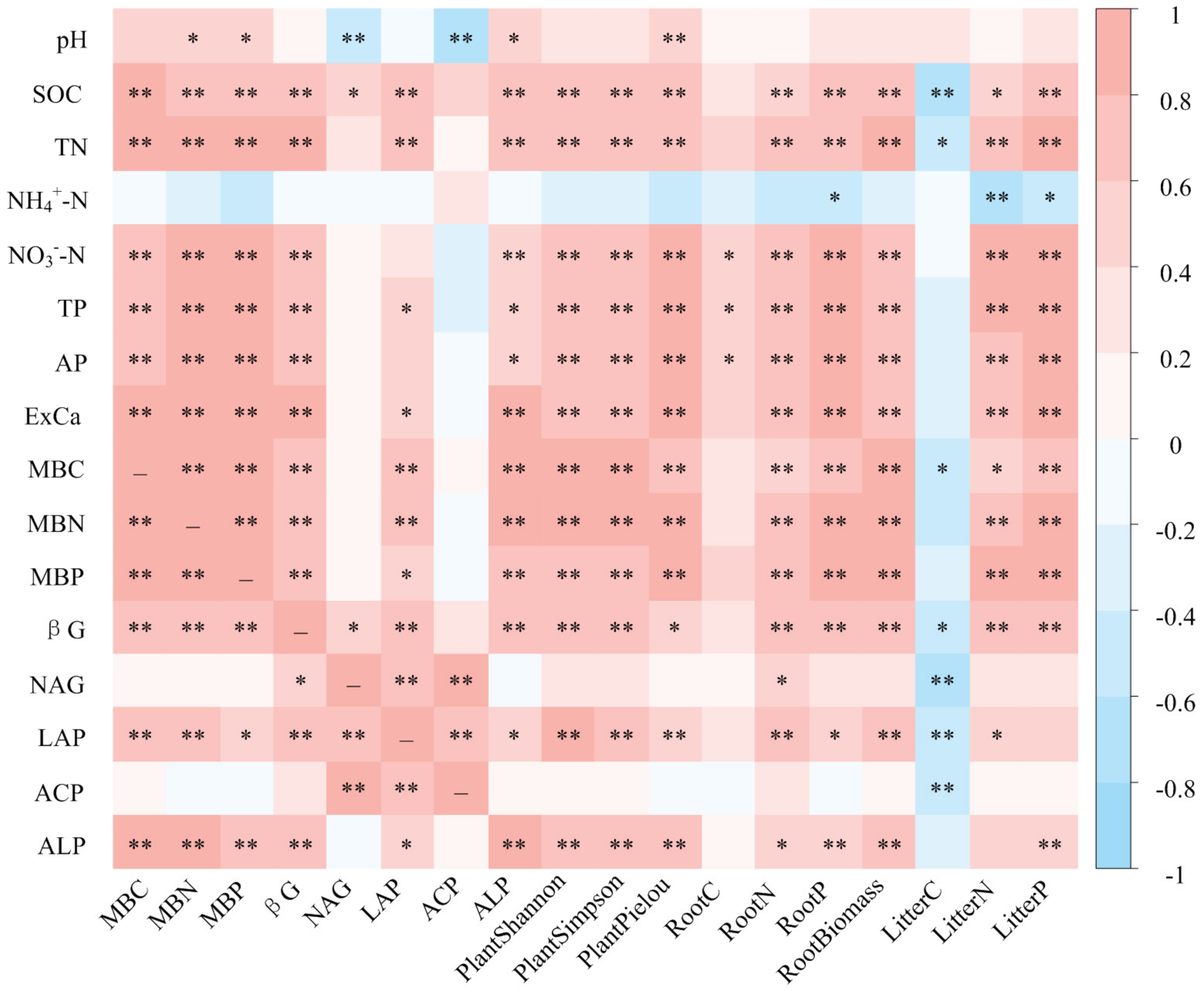

Fig.S1 Soil nutrients, microbial biomass, and enzymes activities related to plant parameters. ** $p < 0.01$; * $p < 0.05$. Root-C, C contents of fine root; Root-N, N contents of fine root; Root-P, P contents of fine root; Litter-C, C contents of litter; Litter-N, N contents of litter; Litter-P, P contents of litter; MBC, microbial biomass C; MBN, microbial biomass N; MBP, microbial biomass P; βG, β-Glucosidase activity; NAG, β-1,4-N-acetylglucosaminidase activity; LAP, Leucine aminopeptidase activity; ACP, Acid phosphatase; ALP, alkaline phosphatase; SOC, soil organic carbon; TN, soil total N; NH_4^+-N, ammonium N; NO_3^--N, nitrate N; TP, soil total P; AP, soil available P. grassland (G), shrubland (SH), shrub-tree land (ST), and forest (F).

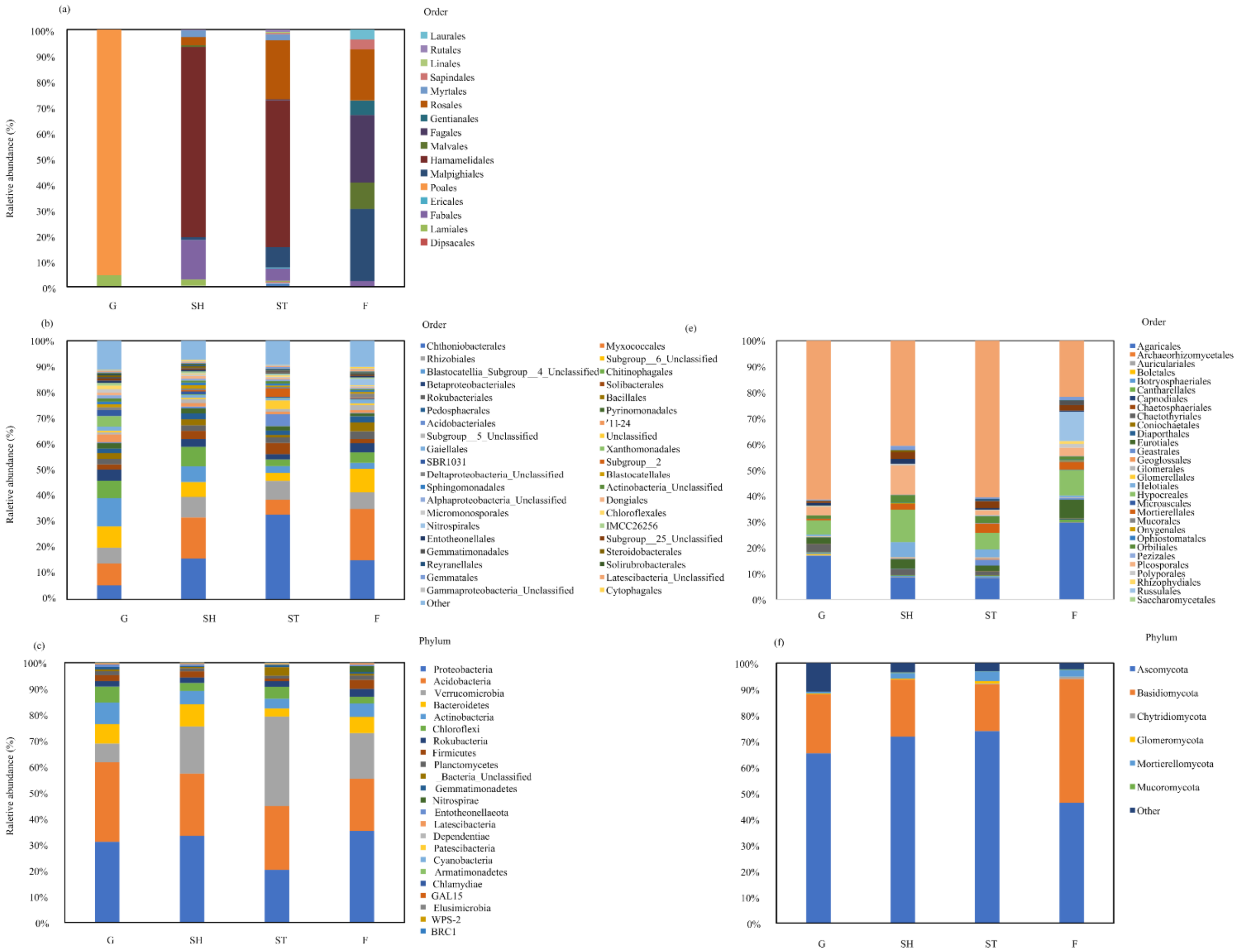

Fig.S2 Relative abundances at Phylum and Order levels of plants (a), bacteria (b, c), and fungi (d, e) in advancing vegetation restorations

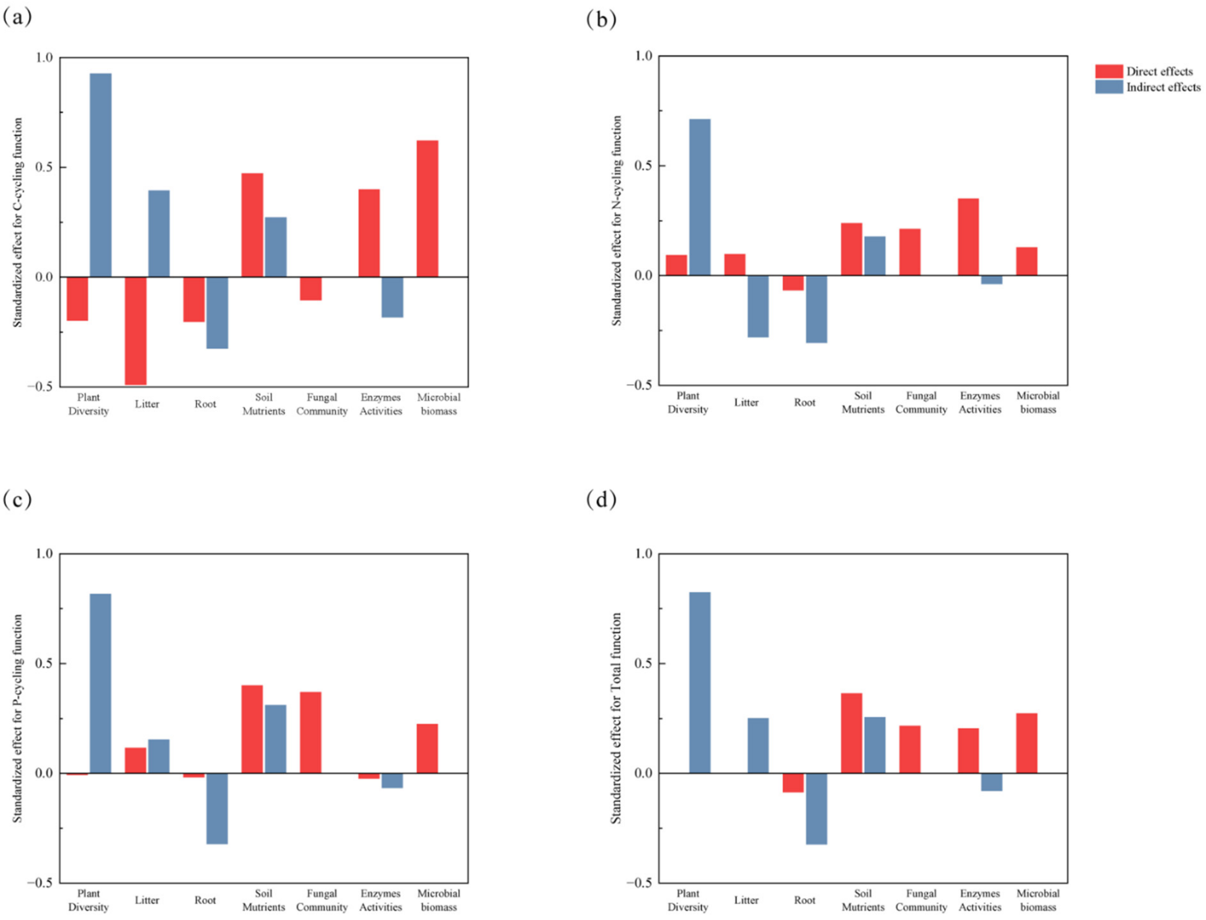

Fig.S3 SEM results showed that the direct and indirect effects of factors on soil C-cycling functional (a), N-cycling functional (b), P-cycling functional (c), and total multifunctional (d) indices

Table S1 Soil pH, soil and litter nutrients, and enzymes activities in advancing vegetation restorations (0~20cm)

Parameters	Grassland	Shrubland	Shrub-tree forest	Forest
pH	6.67±0.09 c	5.82±0.15 a	6.26±0.02 b	6.95±0.14 c
ExCa ($mg.kg^{-1}$)	3.80 ± 0.10 a	3.73 ± 0.21 a	4.80± 0.23 b	6.80 ± 0.51 c
Root biomass ($mg.cm^{-3}$)	1.14 ± 0.06 a	1.83 ± 0.18 b	2.64 ± 0.33 c	3.65 ± 0.27d

ExCa, exchangeable calcium. Lowercase letters indicate significant differences between t stages.

```
### Analysis codes of NMDS in R
bacteriaotu0 = read.csv("bacteria.csv")
head(bacteriaotu0)
group = read.csv("group.csv")
library(vegan)
nmds_bacteriaotu <- metaMDS(bacteriaotu0, distance = 'bray', k = 2)
  summary(nmds_bacteriaotu)
nmds_bacteriaotu$stress (>0.2)
ame(nmds_bacteriaotu$points)
write.csv(nmds_bacteriaotu_site, 'nmds_bacteriaotu_site.csv')
```

```
summary(nmds_bacteriaotu_site)

nmds_bacteriaotu_species <- data.frame(nmds_bacteriaotu$species)
write.csv(nmds_bacteriaotu_species, 'nmds_bacteriaotu_species.csv')

ordiplot(nmds_bacteriaotu, type = 'none', display = 'site',xlim=c(-0.5,0.5),ylim=c(-0.5,0.5),
     main = paste('bacteria, Stress =', round(nmds_bacteriaotu$stress, 4)))
points(nmds_bacteriaotu, display = 'site', pch = group$shape, cex = 0.8,
     col = c(rep('red', 5), rep('green', 5),rep('blue', 5),rep('orange', 5)))
##------------------------------------------
Fungiotu0 = read.csv("Fungi.csv")
head(Fungiotu0)
group = read.csv("group.csv")
library(vegan)
nmds_Fungiotu <- metaMDS(Fungiotu0, distance = 'bray', k = 2)
  summary(nmds_Fungiotu)
nmds_Fungiotu$stress
nmds_Fungiotu_site <- data.frame(nmds_Fungiotu$points)
write.csv(nmds_Fungiotu_site, 'nmds_Fungiotu_site.csv')
summary(nmds_Fungiotu_site)

nmds_Fungiotu_species <- data.frame(nmds_Fungiotu$species)
write.csv(nmds_Fungiotu_species, 'nmds_Fungiotu_species.csv')

ordiplot(nmds_Fungiotu, type = 'none', display = 'site',xlim=c(-1,1),ylim=c(-1,1),
     main = paste('Fungi, Stress =', round(nmds_Fungiotu$stress, 4)))
points(nmds_Fungiotu, display = 'site', pch = group$shape, cex = 0.8,
     col = c(rep('red', 5), rep('green', 5),rep('blue', 5),rep('orange', 5)))
##------------------------------------------
par(mfrow = c(1, 2))
ordiplot(nmds_bacteriaotu, type = 'none', display = 'site',xlim=c(-0.5,0.5),ylim=c(-0.5,0.5),
     main = paste('bacteria, Stress =', round(nmds_bacteriaotu$stress, 4)))
points(nmds_bacteriaotu, display = 'site', pch = group$shape, cex = 1.2,
     col = c(rep('red', 5), rep('green', 5),rep('blue', 5),rep('orange', 5)))
ordiplot(nmds_Fungiotu, type = 'none', display = 'site',xlim=c(-1,1),ylim=c(-1,1),
     main = paste('Fungi, Stress =', round(nmds_Fungiotu$stress, 4)))
points(nmds_Fungiotu, display = 'site', pch = group$shape, cex = 1.2,
     col = c(rep('red', 5), rep('green', 5),rep('blue', 5),rep('orange', 5)))
```
